# Statistical and Computational Methods for Genetic Diseases: An Overview

**DOI:** 10.1155/2015/954598

**Published:** 2015-05-28

**Authors:** Francesco Camastra, Maria Donata Di Taranto, Antonino Staiano

**Affiliations:** ^1^Department of Science and Technology, University of Naples Parthenope, Centro Direzionale Isola C4, 80143 Napoli, Italy; ^2^IRCCS SDN, Via E. Gianturco 113, 80143 Napoli, Italy

## Abstract

The identification of causes of genetic diseases has been carried out by several approaches with increasing complexity. Innovation of genetic methodologies leads to the production of large amounts of data that needs the support of statistical and computational methods to be correctly processed. The aim of the paper is to provide an overview of statistical and computational methods paying attention to methods for the sequence analysis and complex diseases.

## 1. Introduction

The concept that some disease could be inherited by parents was always present, but only after the discovery of DNA as the genetic material, the research about molecular causes of diseases started. Since the first associations of a disease to a defect in a specific gene, the genetic diagnosis becomes an aim of medical scientists in order to early identify the affected patients and to improve their treatments. For simple monogenic diseases, the conventional way to search for mutations in a gene is the sequencing of amplified fragments corresponding to the gene regions. Innovation in molecular methods together with innovation in computational methods allowed developing new analytical techniques useful to unravel most complicated cases. When the gene responsible for the disease is unknown, in order to identify the genetic defects, the next-generation sequencing could be applied to sequence the whole genome/exome of affected patients, producing then a huge amount of data. In early 2001, during the first assemblies of the human genome, Baldi and Brunak, in their seminal book [1], stressed on the need of statistical and computational supports to the genetic analysis: “[…]* these high throughput technologies are capable of rapidly producing terabytes of data that are too overwhelming for conventional biological approaches. As a result, the need for computer/statistical/machine learning techniques is today stronger rather than weaker*”. Today, after fourteen years, the need has become even stronger as the human knowledge of genetic mechanisms still increases, making the research on genetic diseases an amazing adventure as well as difficult and demanding. In case of diseases with a complex etiopathogenesis, for example, those caused by several variants in different genes, more advanced investigations are required. Some examples of methods for association studies are here reported together with methods for meta-analysis of different studies. The study of quantitative traits associated with specific variants is a hot topic in the field of complex diseases, as well as gene expression studies. The presence of a genetic mutation/variant is not the only dysfunction cause of the encoded protein; in fact also alterations in its levels could be responsible for a pathological phenotype. Here we report the example of the combination of both studies, the analysis of expression quantitative trait loci that investigates the association of the quantitative data about gene expression with the presence of specific variants across the genome. Thus the aim of this paper is to provide the reader with an overview of the statistical and computational methodologies, focusing on sequence analysis and complex diseases. Further hot topics, such as methods for next-generation sequencing, gene expression studies, miRNA regulation, and epigenetics, are not discussed merely for sake of space.

The paper is organized as follows: in Section 2, the study of sequence variants is described, while in Section 3 methods for association studies, meta-analysis, and expression quantitative trait loci, specifically targeted to the study of complex diseases, are discussed; finally, some conclusions are drawn in Section 4.

## 2. Sequencing Analysis

The classical approach for identifying the genetic alteration of a hereditary disease is the sequence of causative genes. Although, in the past, a variant identified in patients and not in control subjects was called pathogenic, currently the definition of pathogenicity should be better demonstrated because some variants have only little effects on the disease [2] and could not be considered the real cause of phenotypic alterations. The only one direct criterion to demonstrate the pathogenicity of a variant is the functional characterization of the protein carrying the variant. If this is difficult to be performed, in silico predictions could help.

Research in databases is the fastest way to retrieve information about a variant and to know if the variant was previously identified. The research in database of mutations (e.g., the Human Gene Mutation Database, HGMG—http://www.hgmd.org/) and single nucleotide polymorphisms (SNP) (e.g., http://www.ncbi.nlm.nih.gov/snp) allows linking to previous papers about the variant or linking to 1000 genome data, for example, the variant frequency.

Some mutation types can be immediately considered pathogenic because they lead to a dramatic change of the encoded protein; these include large deletions and insertions comprising one or more exons and deletion and insertion causing reading frameshift and nucleotide substitutions leading to the formation of a premature stop codon (nonsense mutations). Computational predictions are essential for other mutations with uncertain significance, for example, substitutions leading to an amino acid change (missense), not changing amino acid sequence (synonymous), leading to possible splicing alterations and deletion or insertions without frameshift. Different approaches are utilized to evaluate variant effects depending on the mutation type, as listed below.

1. *Missense Mutations*. The change of a single amino acid could not be deleterious if the affected amino acid is not included in the functional domains of the protein or if it is not essential in the protein folding. The simplest method utilized to evaluate the relevance of an amino acid is the multiple alignment of the orthologous sequences allowing identification if the mutated amino acid is conserved during evolution. This is the basis of several algorithms created to evaluate the pathogenicity of a missense mutation such as* SIFT* (Sorting Intolerant From Tolerant; http://sift.jcvi.org/) [3] that is solely based on sequence.* PolyPhen-2* (Polymorphism Phenotyping; http://genetics.bwh.harvard.edu/pph2/) [4] evaluates the variant effect using 11 features based on the sequence alignment and on the structure data selected from a wider pool using machine learning methods. Another tool based on both sequence and structure data is* PMut* (http://mmb2.pcb.ub.es:8080/PMut/) that is based on the use of neural networks [5] trained with disease-associated mutations and neutral variants.* Mutation Taster* (http://www.mutationtaster.org/) [6] is useful for different mutation types and uses 3 different models all based on a Bayes Classifier [5] trained with disease-causing mutations and with neutral polymorphisms.
2. *Synonymous Mutations*. Synonymous mutations are often excluded as causative mutations at the first screening, since they do not cause an apparent change in the protein but they can modify the regulatory mechanisms at the basis of gene expression. Any change in the nucleotide sequence can lead to splicing alterations or to mRNA instability caused by alterations of secondary structure or by altered binding of miRNAs, resulting in decreased protein expression. An additional mechanism of synonymous mutations pathogenicity is due to the alternative codon usage that can increase or decrease the elongation rate depending on the relative abundance of tRNA and influencing the protein folding [7]. Computational approaches to the study of synonymous mutations include the analysis of mRNA structure calculating the ΔG induced by sequence variations [8, 9], of the codon usage [10], of miRNA binding, and of splicing prediction as reported in the next paragraphs.
3. *Splicing*. An intronic nucleotide change near to the acceptor and donor site is easily presumed to affect splicing mechanisms leading to intron retention or exon skipping. Each intronic variant should be assessed for its potential effects on splicing and recently also exonic variants in the CFTR gene leading to a missense variation have been demonstrated to be more relevant in the splicing process than in the protein alteration due to the amino acid change [11]. Tools to identify alterations at the acceptor/donor sites include, for example,* Human Splicing Finder* that calculate the strength of a nucleotide as splicing site based on position weight matrices [12] and* NNSplice* based on a stochastic grammar inference [13].* GeneSplicer* improves splice site detection using an algorithm to characterize the nucleotide sequence around the site based on Markov modeling techniques [14]. Other methods are focused on the evaluation of Exonic Splicing Enhancer such as* ESEfinder* [15].
4. *Deletion or Insertion without Reading Frameshift*. A deletion or an insertion without reading frameshift induces a deletion or an insertion of few amino acids and should be studied with respect to the conservation of involved region and the possible alteration of protein structure. De novo prediction of a protein structure is still a challenge but increasing data of experimentally determined structure allowed creating tools such as* Rosetta* [16] that searches for preexisting structures of fragments with similar sequence and perform the fragment assembly. An innovative approach to the structure study is its coupling with evolution study of protein sequence that help to identify the most important region of the protein [17].

## 3. Complex Diseases

Many common diseases, including heart disease, diabetes, hypertension, and schizophrenia, are complex; that is, they are caused by many genes interacting with environmental factors [18, 19], making its study difficult. Complex diseases are due to the presence of a set of gene variants potentially predisposing to the disease that can develop if other nongenetic factors are present, for example, environmental factors. These diseases are also defined as polygenic and/or multifactorial in order to highlight the complexity of their etiopathogenesis. The genetic variants associated with a complex disease are often common polymorphisms that individually have little impact on the phenotype; for example, the presence of a single variant could not cause any alteration, whereas the presence of several variants in specific conditions could be considered the cause of the disease. In order to determine disease mechanisms, disease-associated genes must be identified and analyzed in combination; nonetheless determining how they interact to cause the disease is a challenge.

### 3.1. Association Studies

First studies on variant associations were conducted by case-control design. In this design, the frequencies of alleles or genotypes at the site of interest are compared in populations of cases and controls; a higher frequency in cases is taken as evidence that allele or genotype is associated with increased risk of disease. The usual conclusion of such studies is that the polymorphism being tested either affects risk of disease directly or is a marker for some nearby genetic variant that affects risk of disease. Due to the modest role of a single variant, the studied population becomes even more large and the number of studied variants increased. Genome-wide association studies (GWAS) have revolutionized human genetics. They have led to the identification of thousands of loci that affect the disease susceptibility and clarified our understanding of the architecture of complex major diseases [20]. In GWAS many common genetic variants in different individuals are analyzed in order to establish if any variant is associated with a phenotypic trait. A* single nucleotide polymorphism*, or SNP, is a single base-pair change in the DNA sequence that occurs with a frequency greater than 1% [21]. Although in the last years a profusion of GWAS for complex human traits was successfully completed [22], even for the simplest analyses there is little general agreement on the most appropriate statistical procedure, including preliminary analyses, that is, Hardy-Weinberg equilibrium testing, inference of phase and missing data, SNP tagging, and single SNP and multipoint tests for association [23]. When a well-defined phenotype has been selected for a study population, and genotypes are collected using well suited techniques, the statistical analysis of genetic data begins. An overview of statistical approaches for genetic association studies is given in [23].

The de facto analysis of genome-wide association data is a series of single locus statistic tests where each SNP is independently examined for association to the phenotype. The usual approach to assess evidence for an association between genetic variants and a phenotype is to compute a *p*-value for the null hypothesis (*H*_0_), of no association. We recall that the *p*-value is the probability of obtaining a result of a statistic test identical to the one actually observed when the null hypothesis is true. Some widely used methods for computing *p*-values are linear regression, logistic regression, Fisher exact test, and *χ* ^2^ test [23, 24]. If multiple tests are performed, adjustments of *p*-values are required. To this aim, several methods are available, for example, Bonferroni, False Detection Rate (FDR), and *q*-value. We recall that the *q*-value of an hypothesis is the minimum FDR at which the test is statistically significant. *q*-values are usually derived from the full distribution of *p*-values across all tests. However, with *p*-value only, it is difficult to quantify how much confident one should be that a given SNP is truly associated with a phenotype. Indeed, the same *p*-value computed at different SNPs or in different studies can have different implications for the plausibility of a true association depending on the factors that affect the power of the test, such as the minor allele frequency of the SNP and the size of the study. This is because the probability that a SNP with a given *p*-value is truly associated with the phenotype depends not only on how unlikely that *p*-value is under *H*_0_ but also on how unlikely it is under the alternative hypothesis *H*_1_ (which differs from test to test) [25]. Bayesian methods provide an alternative approach for assessing associations that alleviates the limitations of *p*-values at the cost of some additional modelling assumptions. As an example, a bayesian analysis requires explicit assumptions about effect sizes at truly associated SNPs. Bayesian methods [5] compute measures of evidence that can be directly compared among SNPs within and across studies, and for combining results across studies, across SNPs in a gene, and across gene pathways. For a comprehensive guide to bayesian methods for genetic association studies, refer to [25]. In general, the discovered genetic variants based on univariate analysis account for only a small proportion of the heritability of complex traits [26, 27]. One possible explanation for the “missing heritability” is that testing for association of the phenotype with each SNP individually is not well suited for detecting multiple variants with small effects [28]. Analyzing SNPs one by one can neglect information on their joint distribution. Therefore, a number of association tests involving multiple SNPs have been applied or developed [23, 29]. The development of a multiple testing procedure involves two steps: ranking the hypotheses and choosing a cutoff (i.e., a threshold value) along the rankings. Different methods use SNPs dependency for choosing the cutoff [30, 31], while [29] uses the dependency of adjacent SNPs, discovered by a* Hidden Markov Model* (HMM) [32], to create more efficient rankings. A gene-based test for association has been, instead, proposed in [33], where a* greedy* [34] bayesian model selection is used to identify the independent effects within a gene and then combined to generate a stronger statistical signal. A further strategy to uncover the “missing heritability” is to use Gene Set Analysis (GSA) as a way to extract additional information from genome-wide SNP data [35]. GSA has the objective of assessing the overall evidence of variant association in a whole set of genes with a disease status. A gene set is a predefined set of genes based on criteria other than the data being analyzed, for example, genes within a specific biological pathway [22]. Several methods for performing the gene enrichment in GSA are based on Fisher's exact test and the *χ* ^2^ test [36]. GSA has the potential to detect subtle effects of multiple SNPs in the same gene set that might be missed when assessed individually [37]. Since numerous genes can be combined into a limited number of gene sets for analysis, the multiple testing burden may be greatly reduced by GSA. Moreover, the incorporation of biological knowledge in the statistical analysis may aid the researchers in the interpretation of the results. For a state-of-the art review of gene set studies the reader can refer to [22], while a thorough review of statistical approaches for “prioritizing” the GWAS results is given in [35]. In [38], instead, the SNPs are grouped into SNP sets on the basis of proximity to genomic features such as gene or haplotype blocks, and then the joint effect of each SNP set is tested. The testing of each SNP set is made via the logistic kernel-machine based test. The latter test provides a statistical framework that allows flexible modeling of epistatic and nonlinear SNP effects ([38] and the references therein). Several further proposals to GWAS come from the machine learning research field [39, 40]. From this perspective, it is argued that methods like* Neural Networks* (NNs) [5],* Support Vector Machine* (SVM) [41], and* Random Forests* (RFs) [42] may more naturally and effectively deal with the high dimensionality of data and the occurrence of multiple polymorphisms with respect to more traditional statistical techniques [40, 43]. A number of applications of NNs and hybrid NN have been developed to study childhood allergic asthma [44], Parkinson's disease [45], Alzheimer's disease [46], and multiple sclerosis [47]. SVM has been applied to Parkinson disease [48] and type 2 diabetes [49], while RFs have been applied to study Crohn disease [50], familial combined hyperlipidemia [51], and colon and ovarian cancers [52].

### 3.2. Methods for Meta-Analysis

To date, a huge number of association studies identified many genetic variants associated with complex diseases. However, these studies often explain only a small proportion of the disease trait's variability [53, 54]. Genetic effects due to common alleles are small and detecting signal requires larger sample sizes [55]. With this growth in evidence has come an increasing need to collate and summarize the evidences in order to identify true genetic associations among the large volume of false positives ([54] and references therein). Furthermore, replication of findings in independent data sets is now widely regarded as a prerequisite for convincing evidence of association [56]. This is why meta-analysis has become an ever more popular approach for the validation of genetic loci predisposing for common disease and phenotypes.* Meta-analyses* can be defined as the statistical integration of information from multiple independent studies with the aim of obtaining an overall estimator (e.g., significance level, *p*-value, and odd ratio) of the investigated association [57]. Most genetic risk variants discovered in the past few years have come from large-scale meta-analyses of GWASs and several hundred GWAS meta-analyses have already been published [58, 59]. Most of these meta-analyses had sample sizes in the discovery phase exceeding 10,000 participants [60]. These efforts have dramatically increased the yield of discovered and validated genetic risk loci and large meta-analyses may continue to increase the yield of loci in proportion to the total sample sizes [57]. GWAS meta-analysis can be organized in a number of stages (see references [58, 59] for a more detailed description and reference [57] for a more concise one). However, this overview is focused on the state-of-the-art of statistical models for data synthesis in GWAS meta-analysis and following closely the review given in [57].

One possible approach, that is, the Fisher's approach [57], is based on combining *p*-values. Here the null hypothesis that the true effect is null in each of the combined data sets is checked against the alternative hypothesis that there is nonnull association in at least one data set. A closely related approach to *p*-value combination is based on the average of *Z*-values [61]. Although the two methods are correlated, one advantage of the *Z*-score approach, over the Fisher method, is that it takes into account the direction of the effect, and it is rather straightforward to introduce the weights for each study. An alternative and popular approach is fixed effects meta-analysis, used for synthesizing GWAS data and resulting to be very effective for prioritizing and discovering phenotype-associated SNPs [62]. Fixed effects meta-analysis assumes that the true effect of each risk allele is the same in each data set. The inverse variance weighting [56] is the most used model for fixed effects meta-analysis, in which each study is weighted according to the inverse of its squared standard error [58]. Cochran-Mantel-Haenszel [63] approach is a further popular used method in genetics which provides similar results to the inverse variance weighting method [61]. A well known estimator of the between-study variance for the random effect approach is the DerSimonian and Laird estimator (see [57] and references therein). However, this method might be less robust with respect to rare variants [64]. Although random effect models are not adopted in discovery efforts, they are suitable when the goal is to estimate the average effect size of the investigated variant and its uncertainty through different populations, for example, as for predictive purposes [65]. In Han and Eskin [66], a novel random effect method has been suggested to improve discovery power when heterogeneity in effect sizes exists across the studies, differently to traditional random effect models. Bayesian techniques have been also used for GWAS meta-analyses. The Bayes factor [67] has been used by the Wellcome Trust Case Control Consortium, while the Coronary Artery Disease Consortium has estimated the posterior probabilities that a given variant is null [68]. Moreover, bayesian methods have been developed to identify the best inheritance model for variants discovered by GWAS meta-analyses [69] and the polygenic structure of complex diseases [70]. Nevertheless, bayesian models have two main drawbacks. Firstly, they depend on the assumption that the parameters of interest follow a given prior distribution. Secondly, their genome-wide implementation can require a huge computational burden [57].

### 3.3. Expression Quantitative Trait Loci

Quantitative trait locus (QTL) is a DNA region associated with a quantitative phenomenon. In most genetic diseases, quantitative traits are often a measure of the disease severity, such as the lipid levels in a dyslipidemia. Genetic variants could be studied for its capacity to affect these quantitative traits and then to influence the disease severity. Differences in gene expression levels between patients and controls are now recognized as an additional mechanism influencing the development of a complex disease. We are here reporting an example of QTL study based on gene expression levels, the expression Quantitative Trait Locus (eQTL), for example, the study of the effect of a DNA variant on the gene expression. Experimental data from eQTL mapping are mainly formed by a genetic map, marker genotypes, and microarray data extracted by a set of individuals. After the removal of systematic effects, it can obtain measures of gene expression levels. This section does not deal with statistical issues related to a correct eQTL experimental design. To this purpose the reader can refer to [71] and references therein.

eQTL data were used for the identification of the so-called* hot spots* [72], constructing gene networks [73] and the setup of subclasses of clinical phenotypes [74], and shortening the list of candidate genes [75]. All these studies are based on the generation of a list of transcripts and the respective genomic locations these transcripts correspond to. The methods for the eQTL localization are mainly based on usual QTL mapping techniques. A* logarithms of odd* (LOD) score curve is computed for each transcript. LOD score allows comparing the probability of measuring the observed values if two loci are linked with respect to the probability of observing the same values at random. LOD score curve is obtained computing LOD score for all genomic positions. Several approaches have been proposed to control the FDR based on *p*-values and *q*-values [76].

Having said that, in eQTL studies *p*-values (corresponding to the peaks of LOD score curves from each transcript) are used to yield and to control the FDR for a list of transcripts mapping to one location. Since this approach takes into account only LOD score peaks, it cannot be used for transcript mapping to multiple loci [76]. In order to cope with this problem, statistical methods have been designed to control the overall FDR for single and multiple linkage [77, 78]. In particular, an empirical bayesian method to eQTL mapping has been proposed by Kendziorski et al. [78]. The method shares information across transcripts to estimate the posterior probability that each transcript maps to each marker. The method has two different steps. Firstly, transcripts are identified. Then, multiple eQTL are identified using the posterior probability. The method states a genome linked to a trait if its posterior probability of linkage is in the top (100 − *α*) percent of all probabilities for the trait. A typical value for *α* is 5.

After having generated the list of transcripts, the identification of the* hot spots* is usually the next task. Hot spots are genomic regions where there is plenty of transcript maps. The simpler method for identifying the hot spots is the following. For each genomic region, the overall number of mapping transcript is computed. Hot spot candidates are the region whose overall number is ranked among highest ones. Although very simple, the method above can fail if there are several loci with effects whose intensity is not adequately large to be considered statistically significant. A strategy for coping with the problem above has been proposed by Kendziorski et al. [78]. The strategy consists in summing evidence in favor of mapping across every transcript and verifying that the obtained score exceeds a given threshold. Further approaches proposed for the hot spots identification consist in computing profiles averaged across correlated transcripts [79] and profiles from transcripts that are functionally related [72]. After having determined the candidate hot spots, it is necessary to use statistical tests in order to assess the confidence that each spot is hot. Therefore a crucial problem is the identification of the so-called* ghost hot spots*, that is, candidate spots that have been considered erroneously hot. This problem has been partially addressed by a Poisson-based test [80] that can detect ghost spots, by computing the probability that a particular genome region would have at least *k* transcripts linked to it if there were not any hot spots. Unfortunately, this test cannot be applied when the candidate hot spots are identified by summing the evidence of linkage across all transcripts.

The detection of hot spots yields list of comapping transcripts and involves the inspection of further candidates controlling the whole collection. This is motivated by the observation that comapping is the result of comembership in a biological pathway where functional information is deduced by means of temporally correlated transcripts. Jansen and Nap [81] showed first how spot list could be used to make networks, represented mathematically by* graphs*. A graph is a couple of a set of vertices and a set of edges, connecting couples of vertices. In this case, a vertex represents either a gene or a transcript. An edge connects two vertices when there is some relationship between them; besides, a weight, measured by correlation coefficient, is generally associated to the edge. Pairwise correlations among all transcripts are used to identify* cliques* [82], namely sets of vertices, representing transcripts, completely connected by edges. We have to recall that the clique's identification in a graph is a NP-problem [34]. This implies that it is an intractable problem if the graph of the transcript is not adequately small. Mapping regions common to clique members are studied to identify potential candidates that are likely affecting the pathway.

Other approaches that can permit the identification of potentially causal relationships among transcripts are the ones based on* bayesian networks* [83]. Bayesian networks have the aim of finding the so-called* best model*, namely, the model that optimally describes the data (i.e., the transcript and/or the loci) in some given model space. Finding the best model usually requires the computation of penalized likelihood that manages the trade-off between the goodness of the fit of the model and the number of model parameters. In order to guarantee that the problem is computationally feasible, the model space has to be moderate. Narrowing down the model space for eQTL mapping is usually performed considering only the transcripts that maps to at least one location [84, 85].

We conclude the section quoting that several software tools for eQTL analysis are currently available [86–88].

## 4. Conclusions

In the paper an overview of statistical and computational methods focused on sequence analysis and complex diseases has been presented. Among the different techniques discussed in this overview, bayesian techniques seem to be promising in terms of performance in some fields, for example, complex diseases [89]. Since these methods generally require a remarkable computational burden, their application has not been popular in the past. Therefore, the development of new high performing computing platforms makes possible, in the next future, a massive use of bayesian techniques in order to cope with biological problems and in particular with complex disease tasks. Although some biological problems have been solved, new ones, even more complex, arise representing, in this way, novel challenges for either biological or statistical and computational methods.
